# A Fast Strategy for Determination of Vitamin B_9_ in Food and Pharmaceutical Samples Using an Ionic Liquid-Modified Nanostructure Voltammetric Sensor

**DOI:** 10.3390/s16060747

**Published:** 2016-05-24

**Authors:** Fatemeh Khaleghi, Abolfazl Elyasi Irai, Roya Sadeghi, Vinod Kumar Gupta, Yangping Wen

**Affiliations:** 1 The Health of Plant and Livestock Products Research Center, Mazandaran University of Medical Sciences, Sari 4815733971, Iran; 2 Young Researchers and Elite Club, Ayatollah Amoli Branch, Islamic Azad University, Mazandaran 46351-43358, Iran; ab.elyasi.ir@gmail.com; 3 Department of Physics, Science and Research Branch, Islamic Azad University, Mazandaran 1477893855, Iran; r00sadeghi@gmail.com; 4 Department of Chemistry, Indian Institute of Technology Roorkee, Roorkee 247667, India; 5 Department of Applied Chemistry, University of Johannesburg, Johannesburg 3204/6, South Africa; 6 Key Laboratory of Applied Chemistry, Jiangxi Agricultural University, Nanchang 330045, China; wenyangping1980@gmail.com

**Keywords:** vitamin B_9_, ZnO/CNTs, ionic liquids, modified electrode

## Abstract

Vitamin B_9_ or folic acid is an important food supplement with wide clinical applications. Due to its importance and its side effects in pregnant women, fast determination of this vitamin is very important. In this study we present a new fast and sensitive voltammetric sensor for the analysis of trace levels of vitamin B_9_ using a carbon paste electrode (CPE) modified with 1,3-dipropylimidazolium bromide (1,3-DIBr) as a binder and ZnO/CNTs nanocomposite as a mediator. The electro-oxidation signal of vitamin B_9_ at the surface of the 1,3-DIBr/ZnO/CNTs/CPE electrode appeared at 800 mV, which was about 95 mV less positive compared to the corresponding unmodified CPE. The oxidation current of vitamin B_9_ by square wave voltammetry (SWV) increased linearly with its concentration in the range of 0.08–650 μM. The detection limit for vitamin B_9_ was 0.05 μM. Finally, the utility of the new 1,3-DIBr/ZnO/CNTs/CPE electrode was tested in the determination of vitamin B_9_ in food and pharmaceutical samples.

## 1. Introduction

Interest in voltammetric sensors for fast analysis has increased in the recent years [1,2,3,4,5], but the high overvoltage and low electrical signal of electroactive compounds, especially in food, pharmaceutical, biological and environmental samples, is problematic for the application of voltammetric sensors [6,7,8,9,10,11,12]. Modified electrodes have been used as voltammetric sensors with good ability for trace level analysis [13,14,15,16,17,18,19,20]. Ionic liquids (ILs) have some unique properties, such as a low vapor pressure, good thermal stability, high polarity, tunable viscosity and an ability to dissolve many compounds, a wide electrochemical window, high conductivity, high heat capacity and they can act as solvents available to control reactions. ILs represent a new class of conductive binders and mediators for the modification of electrodes for trace analysis [21,22,23,24]. In general, the structure of an IL and its high conductivity at a surface of electrodes are extremely important for evaluating and selecting ionic liquids, especially room temperature ionic liquids, for electrochemical applications [25,26,27,28,29,30].

Nanotechnology and nanoscience represent new and enabling platforms that promise to provide a broad range of novel uses and improved technologies for environmental, biological and other scientific applications [31,32,33,34,35,36,37,38,39]. Nanomaterials have been another acceptable choice for the modification of electrochemical sensors in recent years [40,41,42,43,44,45]. These kinds of materials display high conductivity and have been used in different fields to increase the efficiency of electrochemical sensors in trace level analysis [46,47,48].

Vitamin B_9_ (folic acid) is a water soluble vitamin that is very important for the production and maintenance of new cells. In the human body it is necessary for make normal red blood cells and to prevent anemia [49]. There is also some evidence that sufficient folic acid in the diet can reduce the risk of heart disease, although this evidence is based on population studies and not on more definitive clinical trials, so there is no definitive evidence that taking folic acid supplements would help in this respect.

Many analytical methods have been reported for the analysis of vitamin B_9_, including electrochemical sensors [50,51,52], spectrophotometry [53], chemiluminescence [54], Capillary electrophoresis [55] and HPLC [56]. In this project, we have developed a simple and fast method for the synthesis of ZnO/CNTs nanocomposite and its application for the preparation of electrochemical sensors in the presence of 1,3-DIBr as a high conductive IL binder. Next, the analytical performance of the novel 1,3-DIBr/ZnO/CNTs/CPE electrode was checked in the square wave voltammetric electro-oxidation of vitamin B_9_ in food and pharmaceutical samples. The obtained results showed the superiority of 1,3-DIBr/ZnO/CNTs/CPE over unmodified electrodes in terms of better reversibility and higher sensitivity.

## 2. Experimental Section

### 2.1. Apparatus and Chemicals

Vitamin B_9_ (>97%) was obtained from Sigma-Aldrich (CAS Number 59-30-3, St. Louis, MS, USA) and graphite powder (<50 µm) and paraffin oil for the preparation of carbon paste electrode were obtained from Merck (Darmstadt, Germany). A stock 0.001 M solution of vitamin B_9_ was prepared by dissolving 0.015 g vitamin B_9_ in 100 mL of buffer solution. Phosphate buffer solutions (PBS) with different pH values were used for optimization of pH. Square wave and linear sweep voltammetric investigation were performed using a μ-Autolab potentiostat/galvanostat (Eco Chemie, Utrecht, The Netherlands) connected to a three-electrode cell. An Ag/AgCl/KCl_sat_ electrode, a platinum wire, and the novel 1,3-DIBr/ZnO/CNTs/CPE electrode were used as the reference, auxiliary and working electrodes, respectively. Scanning electron microscopy (KYKY-EM3200 Digital Scanning Electron Microscope, KYKY Technology Development Ltd., Beijing, China) was used for morphological investigation. X-ray powder diffraction studies were carried out using a STOE diffractometer (EQuniox 3000, Inel, France) with Cu-Kα radiation (k = 1.54 Å). ZnO/CNTs and 1,3-DIBr were synthesized according to previous published papers [17,57].

### 2.2. Preparation of the Electrode

1,3-DIBr/ZnO/CNTs/CPE was prepared by mixing 1,3-dipropylimidazolium bromide (1,3-DIBr 0.25 g), paraffin oil (0.75 g), ZnO/CNTs (0.1 g), and graphite powder (0.9 g). Next, the mixture was mixed well for 75 min until a uniformly wetted paste was obtained. A portion of the paste was firmly pressed into a glass tube to prepare the 1,3-DIBr/ZnO/CNTs/CPE electrode.

### 2.3. Preparation of Real Samples

Mint leaves (6 g) were extracted with 0.1 M pH 9.0 phosphate buffer (100 mL) and 0.1% (*v*/*v*) 2-mercaptoethanol (0.06 g), then the thus obtained mixture was shaken for 50 min in a rotational shaker, and centrifuged at 3500 rpm for 20 min. Finally, the obtained solution filtered with a 47 mm filter (Millipore, Boston, MA, USA). For pharmaceutical analysis, five commercial vitamin B_9_ tablets (50.0 mg per tablet) were completely ground and homogenized. Next, suitable amounts of the powders was accurately weighed and dissolved in 100 mL of buffer solution, and the mixture was filtered through a 42 mm filter. Fortified juice samples were obtained from local supermarkets and then centrifuged for 20 min at 2000 rpm. The supernatant was filtered using a 42 mm filter and the filtrate used for the real sample analysis.

## 3. Results and Discussion

### 3.1. ZnO/CNTs Characterization

**Figure 1:**
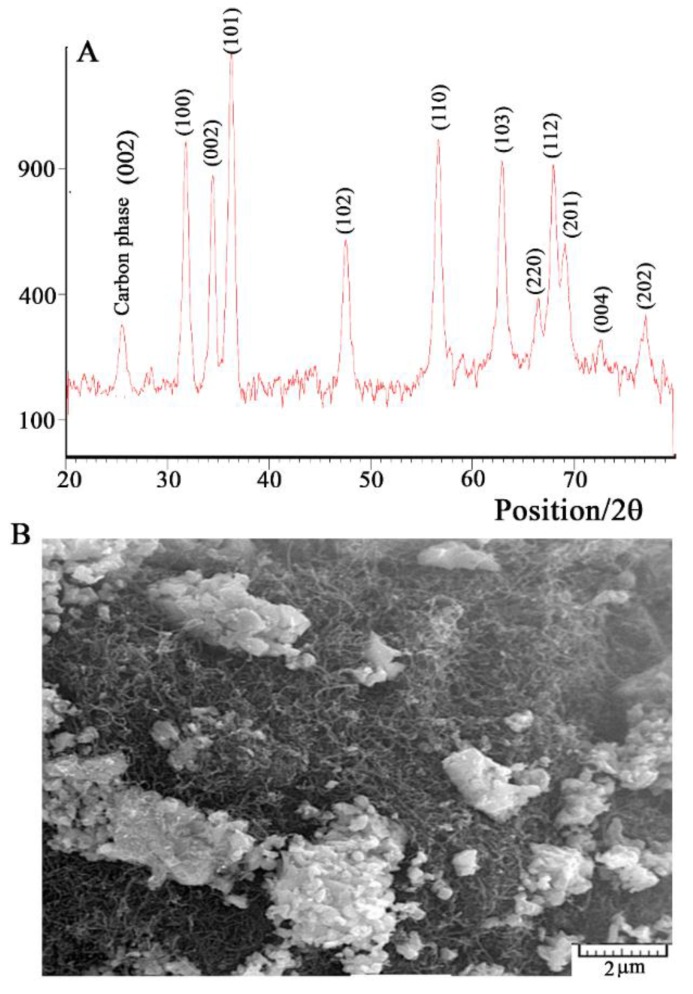
(**A**) XRD pattern of ZnO/CNTs nanocomposite; (**B**) SEM images of ZnO/CNTs nanocomposite.

Figure 1A shows the XRD pattern for the synthesized ZnO/CNTs nanocomposite over a 2θ range of 20°–80°. XRD pattern (Figure 1) confirmed that the synthesized materials were ZnO [56]. The peak at ~26 can be nicely indexed to the (002) plane of CNTs as marked with star in Figure 1A. Also, as can be seen in Figure 1B, the outside surface of the carbon nanotubes is uniformly dotted with ZnO nanostructures, which is in agreement with results obtained from the XRD pattern.

### 3.2. Voltammetric Investigation

**Figure 2:**
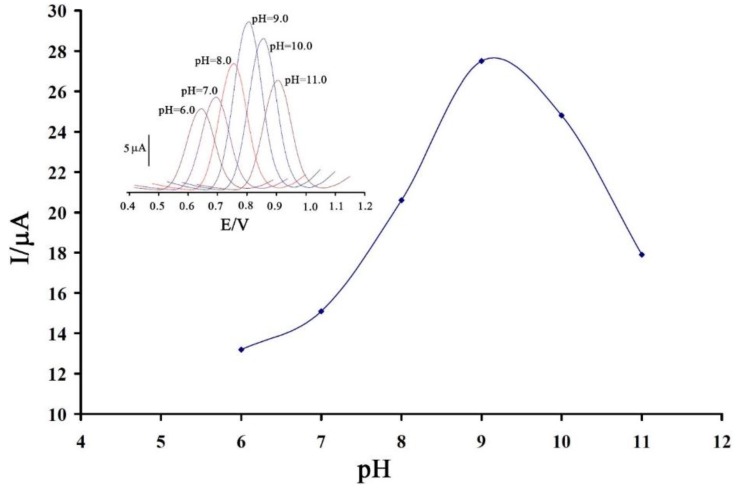
Plot of peak current (I_pa_) *vs.* changing in pH value for the electro-oxidation of 100 µΜ vitamin B_9_ at a surface of 1,3-DIBr/ZnO/CNTs/CPE. Inset: influence of pH on square wave voltammograms of vitamin B_9_ at the surface of the modified electrode.

In the first step we investigated the effect of pH on the electro-oxidation of vitamin B_9_ using the SWV technique (Figure 2 insert). As can be seen, the vitamin B_9_ peak current increased regularly from pH 6.0 to 9.0, and then conversely the current decreased when the pH value increased further from 9.0 to 11.0 (Figure 2). Therefore pH 9.0 was chosen as the optimal experimental condition for other experiments. The relationship between the oxidation peak potential and pH was also determined.

**Figure 3:**
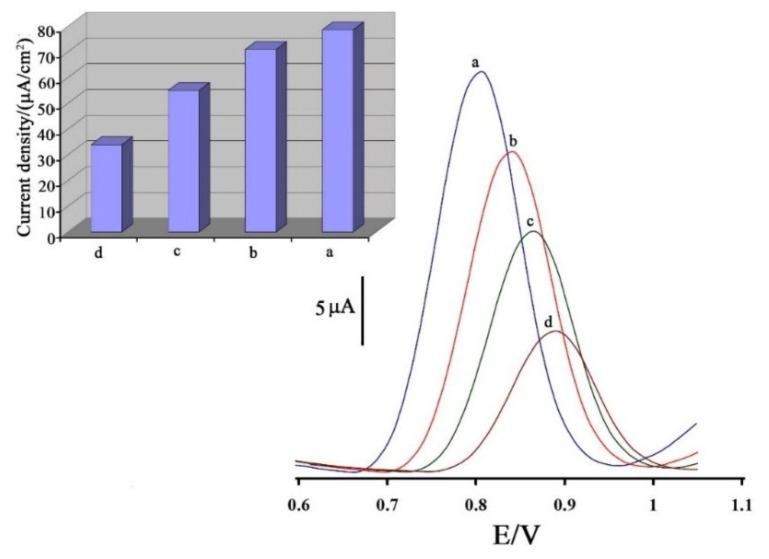
Square wave voltammograms of (**a**) 1,3-DIBr/ZnO/CNTs/CPE; (**b**) 1,3-DIBr/CPE; (**c**) ZnO/CNTs/CPE and (**d**) CPE in presence of 100 μM vitamin B_9_ at pH 9.0, respectively. Inset: The current density derived from square wave voltammograms responses of 100 μM vitamin B_9_ at pH 9.0 at the surface of different electrodes.

A linear shift of E_pa_ towards negative potential with increasing pH can be obtained, which fitted the regression equation E_pa_ (V) = −0.057pH + 0.985, indicating that protons are directly involved in the oxidation of vitamin B_9_. A slope of 57 mV/pH suggests that the number of electrons transferred is equal to the number of protons involved in the electrode reaction [39]. The current density results are shown in the insert of Figure 3 for different electrodes. The results confirmed that the joint presence of ZnO/CNTs and 1,3-DIBr causes an increase in the electrode conductivity. In order to establish a good sensitivity and highly selective electrochemical sensor for the detection of vitamin B_9_ with ZnO/CNTs and 1,3-DIBr as the electron mediators, we first investigated the voltammetric behavior of vitamin B_9_ at the surface of different electrodes. The results indicated that the oxidation peak currents of vitamin B_9_ at 1,3-DIBr/ZnO/CNTs/CPE can be significantly enhanced, so it’s replacement for bare CPE, ZnO/CNTs/CPE and 1,3-DIBr/CPE was subsequently exploited as an electrochemical sensor for effective sensing of vitamin B_9_. As also seen from this figure, the electro-oxidation peak potential of vitamin B_9_ at the surface of the 1,3-DIBr/ZnO/CNTs/CPE appeared at 800 mV, which was about 95 mV lower than the oxidation peak potential at the surface of the bare CPE under similar conditions. At the same time, the electro-oxidation peak current was increased by ~2.83 times at the 1,3-DIBr/ZnO/CNTs/CPE surface compared to CPE.

**Figure 4:**
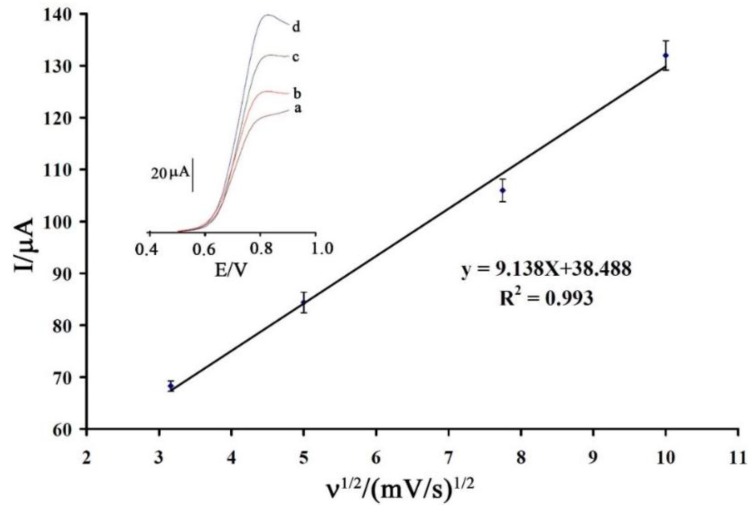
Plot of I_pa_ *vs.* ν^1/2^ for the oxidation of vitamin B_9_ at 1,3-DIBr/ZnO/CNTs/CPE. Inset shows linear sweep voltammograms of vitamin B_9_ at 1,3-DIBr/ZnO/CNTs/CPE at different scan rates of (**a**) 10; (**b**) 25; (**c**) 60 and (**d**) 100 mV/s in 0.1 M phosphate buffer, pH 9.0.

Figure 4 shows the effect of scan rate (υ) on the electro-oxidation of vitamin B_9_ under the optimum conditions. The results show that the peak current increased linearly as the square root of scan rate increased over a range of 10 to 100 mV/s. This result shows that the electrode process for oxidation of vitamin B_9_ is controlled by a diffusion step.

**Figure 5:**
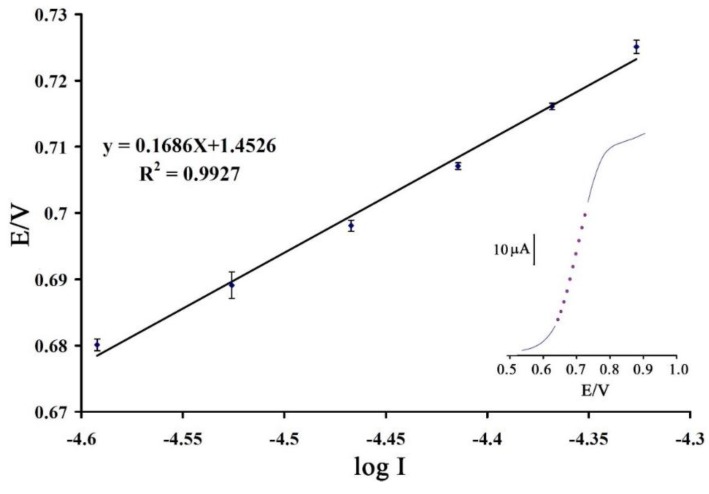
Tafel plot for 1,3-DIBr/ZnO/CNTs/CPE in 0.1 M PBS (pH 9.0) with a scan rate of 10 mV/s in the presence of vitamin B_9_.

The value of α was obtained from a Tafel plot (Figure 5). The slope of the Tafel plot is equal to 2.3RT/*n* (1 − α) F which comes up to 0.1686 Vdecade^−1^. We obtained α as 0.82. On the other hand, we obtained the value of (α) 0.22 at a surface of a bare electrode. These values clearly show that not only is the overpotential for vitamin B_9_ oxidation reduced at the surface of 1,3-DIBr/ZnO/CNTs/CPE, but also the rate of the electron transfer process is greatly enhanced, a phenomenon confirmed by the larger I_pa_ values recorded during the voltammetric responses at 1,3-DIBr/ZnO/CNTs/CPE.

**Figure 6:**
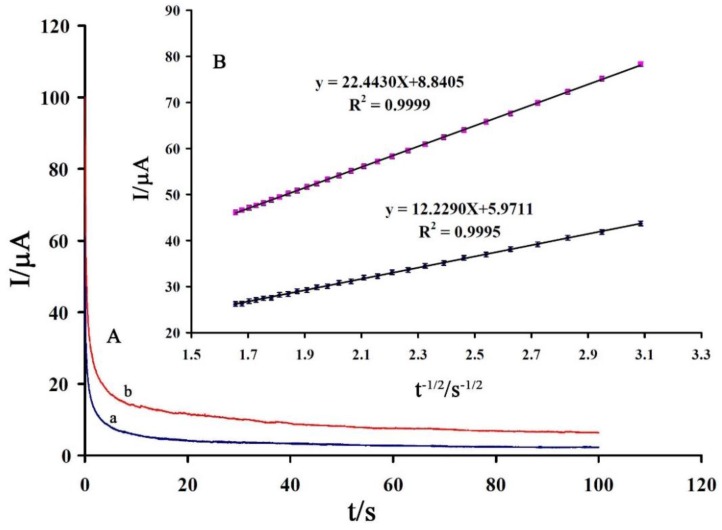
(**A**) Chronoamperograms obtained at 1,3-DIBr/ZnO/CNTs/CPE in the presence of (line a) 300 and (line b) 400 μM vitamin B_9_ in the buffer solution (pH 9.0); (**B**) Cottrell plot for the data from the chronoamperograms.

A chronoamperometric method was used for determination of the diffusion coefficient (D; can be obtained from slope of Cottrell plots) of vitamin B_9_ using the data derived from the raising part of the current-voltage curve (Figure 6A). From the result of Figure 6B and the Cottrell equation the mean value of the D was found to be 1.65 × 10^−6^ cm^2^/s.

### 3.3. Analytical Parameters for Determination of Vitamin B_*9*_

SWV was used for the sensitive determination of vitamin B_9_ at the 1,3-DIBr/ZnO/CNTs/CPE electrode because of its higher current sensitivity. The quantitative evaluation was based on a linear correlation between the peak currents and the concentration added, resulting in a good correlation. The equation for the measurement of vitamin B_9_ was Ip(A) = 0.2058 C + 4.2628 (in the range 0.08–650 μM) with a correlation coefficient of *R*^2^ = 0.9975. The detection limit was determined at 0.05 μM vitamin B_9_ according to the definition of Y_LOD_ = Y_B_ + 3σ. This value of the detection limit and the linear dynamic range for vitamin B_9_ observed on the 1,3-DIBr/ZnO/CNTs/CPE electrode are comparable and even better than those obtained for other modified electrodes (Table 1).

### 3.4. Stability and Reproducibility

**Figure 7:**
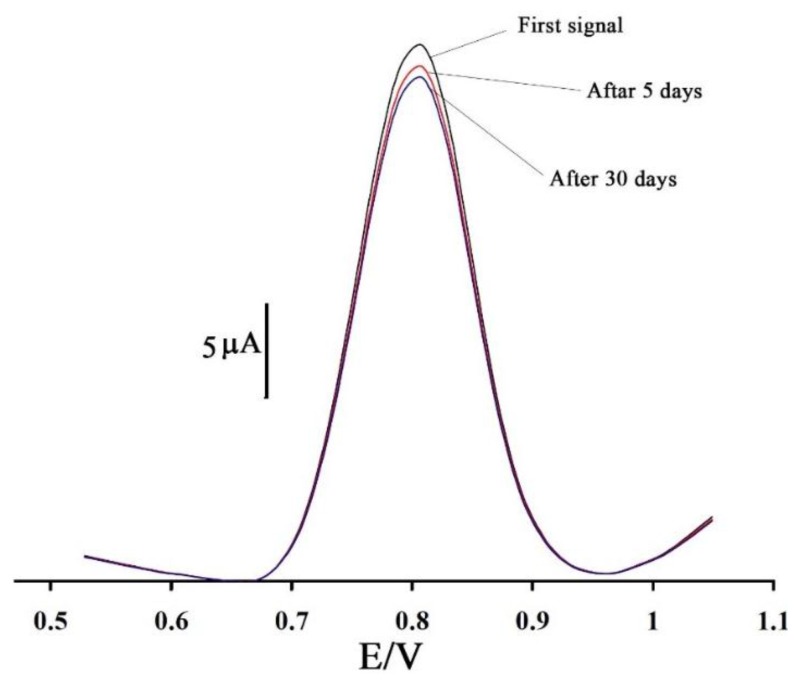
Square wave voltammograms of 1,3-DIBr/ZnO/CNTs/CPE in the presence of 100 μM vitamin B_9_ at a pH 9.0 at a different times.

The repeatability and stability of 1,3-DIBr/ZnO/CNTs/CPE was investigated by square wave voltammetry measurements of 5.0 µM vitamin B_9_. The relative standard deviation (RSD%) for eleven successive assays was 1.5%. When using ten different electrodes, the RSD% for eleven measurements was 2.1%. When the 1,3-DIBr/ZnO/CNTs/CPE electrode is stored in the laboratory, it retains 96% of its initial response after 5 days and 94% after 30 days (Figure 7). These results indicate that 1,3-DIBr/ZnO/CNTs/CPE has good stability and reproducibility, and could be used for vitamin B_9_ analysis.

### 3.5. Interference Study

For a successful voltammetric sensor for the detection of vitamin B_9_ in food and pharmaceutical samples, good selectivity and high sensitivity are the two most important requirements. To assess the selectivity of the 1,3-DIBr/ZnO/CNTs/CPE, some potential interferents were investigated in the presence of 10.0 μM vitamin B_9_. As can be seen in Table 2, the 1,3-DIBr/ZnO/CNTs/CPE has a good selectivity for the determination of vitamin B_9_.

### 3.6. Real Sample Analysis

For the purposes of this study, vitamin B_9_ was electrochemically measured with the developed voltammetric sensor in vitamin B_9_ tablets and food samples. The data obtained by the proposed method was compared with a published method too [39] (see Table 3; a modified carbon paste electrode prepared with *N*-hexyl-3-methylimidazolium hexafluorophosphate and Pt:Co was used for comparing the obtained data). As can be seen from Table 3, the amounts of vitamin B_9_ determined by our 1,3-DIBr/ZnO/CNTs/CPE electrode in real samples were very similar to the labelled amount and there was no difference at the 95% confidence level (paired t test; n = 3) when compared with the published method [39]. Therefore, the 1,3-DIBr/ZnO/CNTs/CPE is very suitable for the voltammetric determination of vitamin B_9_ in real samples.

## 4. Conclusions

In the present work, the combination of the features of ZnO/CNTs nanocomposite and 1,3-dipropylimidazolium bromide were exploited for the development of a voltammetric sensor for the determination of vitamin B_9_. The developed voltammetric sensor based on carbon nanotubes-ionic liquid composite was shown to be simple, quick to prepare, reproducible, stable and precise for the voltammetric determination of vitamin B_9_. The new 1,3-DIBr/ZnO/CNTs/CPE electrode was successfully used for the determination of vitamin B_9_ in some food and pharmaceutical samples.

## Figures and Tables

**Table 1:** Comparison of the efficiency of some electrochemical methods in the determination of vitamin B_9_.

Method	Electrode	Modifier	pH	LOD ^a^	LDR ^b^	Reference
DPV ^c^	GCE ^d^	SWCNT/IL ^e^	5.5	0.001	0.002–1.0	[52]
Cyclic voltammetry	Au	MBT/SAM ^f^	7.4	0.004	0.008–1.0	[58]
SWV	CPE	ZnO/NPs/IL	9.0	0.01	0.05–550	[50]
SWV	CPE	Pt:Co nanoalloy/IL	9.0	0.04	0.1–500	[39]
SWV	CPE	Gold nanoparticles	6.5	0.0027	0.006–80	[59]
SWV	CPE	1,3-DIBr/ZnO/CNTs	9.0	0.05	0.08–650	This work

**^a^** Limit of detection; ^b^ linear dynamic range; ^c^ Differential pulse voltammetry; ^d^ Glassy carbon electrode; ^e^ Single-walled carbon nanotubes; ^f^ 2-mercaptobenzothiazole self-assembled gold electrode.

**Table 2:** Interference study for the determination of 10.0 µM vitamin B_9_.

Species	Tolerance Limits (W_Substance_/W_vitamin_ B9)
Glucose, leucine, glycine, methionine, alanine, valine, histidine	900
Uric acid and ascorbic acid, vitamin B_6_	400
Starch	Saturation

**Table 3:** Determination of vitamin B_9_ in real samples (n = 3).

Sample	Found (Vitamin B_9_) Proposed Method (μM)	Found (Vitamin B_9_) Other Method (μM)	F_ex_	F_tab_	t_ex_	t_tab_
Tablet	10.22 ± 0.55	9.22 ± 0.65	7.8	19.0	1.5	3.8
Mint vegetable	4.87 ± 0.25	5.32 ± 0.35	6.7	19.0	1.1	3.8
Orange juice	14.98 ± 0.75	15.78 ± 0.95	12.9	19.0	2.6	3.8
Apple juice	13.42 ± 0.69	12.98 ± 0.75	8.3	19.0	1.8	3.8

F_ex_ is calculated F-value; F_tab_ is the F value obtained from one-tailed table of F-test; t_ex_ is calculated value of t-student test; t_tab_ is the t-value obtained from the table of student t-test.
